# IGF1R Enhances Calcium Oxalate Monohydrate-Induced Epithelial-Mesenchymal Transition by Reprogramming Metabolism via the JAK2/STAT3 Signaling

**DOI:** 10.7150/ijbs.104311

**Published:** 2025-01-01

**Authors:** Jiashan Pan, Yi Zhang, Rui Yao, Mo Yang, Xike Mao, Zhenyu Song, Yuexian Xu, Yang Chen, Bingbing Hou, Xiaoying Liu, Wei Wang, Zongyao Hao

**Affiliations:** 1 Department of Urology, The First Affiliated Hospital of Anhui Medical University, Hefei, China.; 2 Institute of Urology, Anhui Medical University, Hefei, China.; 3 Anhui Province Key Laboratory of Genitourinary Diseases, Anhui Medical University, Hefei, China.; 4 School of Life Sciences, Anhui Medical University, Hefei 230032, China; Henan International Joint Laboratory of Non-Coding RNA and Metabolism in Cancer, Henan Provincial Key Laboratory of Long Non-Coding RNA and Cancer Metabolism, Translational Research Institute of Henan Provincial People's Hospital and People's Hospital of Zhengzhou University, Henan 450053, China.; 5 Department of Urology, Fuyang Hospital of Anhui Medical University, Fuyang 236000, China.

**Keywords:** Calcium Oxalate Monohydrate (COM), Insulin-like Growth Factor 1 Receptor (IGF1R), Glycolysis, Lactate Dehydrogenase A (LDHA), Epithelial-Mesenchymal Transition (EMT)

## Abstract

**Background:** Kidney stone disease is a major risk factor for impaired renal function, leading to renal fibrosis and end-stage renal disease. High global prevalence and recurrence rate pose a significant threat to human health and healthcare resources. Investigating the mechanisms of kidney stone-induced injury is crucial.

**Materials and Methods:** We examined the relationship between insulin-like growth factor 1 receptor (IGF1R) and epithelial-mesenchymal transition (EMT) at three levels: in patients with kidney stones, in mice induced with glyoxalate crystals, and in HK2 cells stimulated with calcium oxalate monohydrate (COM). RNA sequencing (RNA-seq) and untargeted metabolomics were used to investigate IGF1R's biological mechanisms, followed by* in vivo v*alidation in mice.

**Results:** IGF1R was elevated in the kidney stone model, which was significantly associated with EMT progression. RNA-seq analysis indicated that IGF1R enhances EMT through the JAK2/STAT3 pathway. Further experiments at mRNA and protein levels confirmed the activation of this pathway regulated by IGF1R, promoting EMT. Additionally, untargeted metabolomics revealed that IGF1R drives the activation of lactate dehydrogenase A (LDHA) in glycolysis, further facilitating EMT. *In vivo* experiments confirmed that IGF1R increases LDHA activity through the activation of the JAK2/STAT3 pathway, thereby enhancing the EMT.

**Conclusion:** IGF1R promotes EMT in COM-induced kidney injury by activating LDHA via the JAK2/STAT3 signaling.

## Introduction

Nephrolithiasis, commonly known as kidney stones, represents a widespread global health concern with an increasing incidence and recurrence rate, posing a significant public health challenge^1^. Researches indicate that over half of the affected individuals experience recurrence, which is closely associated with severe renal damage and serves as a critical factor leading to chronic kidney disease (CKD) and end-stage renal diseases (ESRD)^2^, ^3^. Further studies reveal that nephrolithiasis is not merely a result of mechanical injury but involves complex metabolic disturbances^4^. Specifically, Calcium Oxalate Monohydrate (COM) are closely related to imbalances in calcium, oxalate, and uric acid metabolism^5^-^7^. These metabolic abnormalities not only induce kidney stone formation but may also exacerbate renal injury, thereby promoting the progression of chronic kidney diseases^8^.

The damage induced by kidney stones may further worsen the condition by promoting metabolic disruptions. Studies have shown that oxidative stress and inflammatory responses caused by kidney stones may trigger epithelial-mesenchymal transition (EMT), a progression critical in chronic fibrosis and cancer progression^9^, ^10^. EMT results in the loss of epithelial cell polarity and cell-cell junctions, transforming them into migratory mesenchymal cells^11^, ^12^. Therefore, it is of great scientific and clinical significance to study whether COM-induced damage triggers EMT progression, and to explore the relationship between COM-induced metabolic disorders and EMT^13^, ^14^.

The insulin-like growth factor 1 receptor (IGF1R) is a critical mediator in metabolic regulation and exerts a profound influence on various metabolic disorders^15^. For instance, IGF1R modulates glucose metabolism through the insulin signaling pathway, thereby playing a pivotal role in insulin resistance and the progression of diabetes^16^. Additionally, IGF1R is implicated in the regulation of liver lipid metabolism in non-alcoholic fatty liver disease (NAFLD), contributing to both fat accumulation and liver fibrosis^17^.

Beyond its role in metabolic diseases, IGF1R is also intricately linked to the EMT progression. In hepatocellular carcinoma (HCC), the overexpression of IGF1R is recognized as a key driver of EMT, significantly enhancing the migratory and invasive capabilities of cancer cells^18^. Similarly, in breast cancer, IGF1R promotes EMT by activating a multitude of signaling pathways, thereby fostering a more aggressive tumor phenotype^19^, ^20^. These findings collectively underscore the multifaceted role of IGF1R in both metabolic regulation and EMT, suggesting that IGF1R may similarly influence EMT in the context of kidney stone formation. However, this intriguing hypothesis necessitates further empirical validation to elucidate the precise mechanisms and clinical implications.

The potential implications of IGF1R in kidney stone-related EMT could extend beyond basic research, offering novel therapeutic targets for the prevention and treatment of kidney stone disease. By understanding the role of IGF1R in EMT, it may be possible to develop targeted interventions that mitigate the progression of kidney stone formation and associated complications. Such advancements could significantly enhance patient outcomes and reduce the burden of kidney stone disease on healthcare systems. In summary, the elucidation of IGF1R's role in metabolic regulation and EMT not only deepens our understanding of these processes but also opens new avenues for therapeutic innovation.

To elucidate the mechanisms by which IGF1R mediates metabolic disturbances and EMT induced by kidney stone damage, we performed a combined analysis of transcriptomics and untargeted metabolomics in a COM-induced cell injury model. Transcriptomics reveals changes in gene expression, while metabolomics reflects alterations in cellular metabolic states. The integration of these two results provides a comprehensive view from genes to metabolites, enabling simultaneous observation of multiple pathways and processes, thus facilitating the discovery of novel mechanisms.

## Materials and methods

### Animal experiments and reagents

The animal study was approved by the Animal Experimentation Ethics Committee of Anhui Medical University (No: LLSC20242203) and conducted following the NIH “Guide for the Care and Use of Laboratory Animals” (8th Edition, 2011). We meticulously selected 6 to 8-week-old male C57BL/6J mice as experimental subjects to ensure the precision and reliability of the experimental outcomes. Mice were acclimatized for 7 days before the experiments.

A double blinded procedure was employed during the experiments, with treatment drugs prepared by researchers not involved in animal handling. Mice were randomly divided into six groups: NC (normal control), NC+Vector, Vector+Glyoxylic acid (Vector+Gly), Gly+Picropodophyllotoxin (PPP), Gly+AAV-shIGF1R, and Gly+AAV-shIGF1R+Colivelin. No mice were excluded from statistical analysis. All procedures follow to the German Animal Welfare Act and followed ARRIVE guidelines^21^. PPP^22^ was administered via intraperitoneal injection at 20 mg/kg every 12h for 14 days, while the control group received saline. AAV9 vectors specifically delivering shIGF1R were used to silence IGF1R expression in mouse kidneys through tail vein injection. That is, the vector, pAAV-NPHS2-EGFP-5'mir30a-shRNA (IGF1R)-3, was dissolved in 200 μL saline at 2.87×10¹³ vgs/mL, with empty vector as control. pAAV-Nphs2-EGFP5'miR30a-shRNA-3'miR30a was at 2.25×10¹³ vgs/mL. The JAK2/STAT3 activator Colivelin (TargetMOI, Cat. # TP1856) was injected continuously for 6 days, starting from the 4th week after the AAV9-shIGF1R injection. Each group consisted of six mice.

After the procedure, mice were euthanized by CO_2_ inhalation, and kidney tissues were collected for subsequent pathological examination, immunohistochemistry, and other tests.

### Acquisition of kidney tissue samples from patients with kidney stone

Kidney tissue containing Randall's plaques was collected from patients undergoing percutaneous nephrolithotomy for kidney stones. Normal kidney tissue was obtained from paracancerous tissue kept in our laboratory (6 pairs). The collection and use of clinical samples were approved by the Ethics Committee of Anhui Medical University, and each patient signed a written informed consent (Number: Quick-PJ2024-05-91).

### Cell culture and reagents

The human proximal tubular epithelial cell line (HK2) was purchased from Pricella Biotechnology Co., Ltd (Cat. #CL-0109). Cells were cultured in Minimum Essential Medium (MEM) (Pricella Biotechnology Co., Ltd, Wuhan, China; Cat. #CM-0109) containing 10% Fetal Bovine Serum (FBS), 100 U/mL penicillin, and 100 μg/mL streptomycin from Life Technologies (Carlsbad, CA, USA) at 37°C in an incubator with 5% CO_2_. Stone model creation: COM (Sigma, Cat. #: C0350000) was added at a concentration of 200 ng/mL. Unless otherwise specified, all cells were cultured for 48h before subsequent treatments.

The siTran 2.0 siRNA transfection reagent (Cat. # TT320002) was purchased from OriGene. The HK-2 cells were stimulated with COM at a concentration of 100 µg/mL for 48h, and then the inhibitor of STAT3, Stattic (TargetMOI, Cat. # T6308), and the activator Colivelin were used at the concentration of 5 μM and 10 μM, respectively (Supplementary Table 5).

### Quantitative real-time reverse transcription - PCR (RT-qPCR) analysis

Total RNA was extracted from the kidney tissues and cells using TRIzol (Gibco, Life Technologies, CA, USA) and reverse transcribed to cDNA using the iScript cDNA Synthesis Kit (Bio-Rad, USA). Real-time quantitative PCR (qPCR) analysis was then performed using the Bio-Rad iQ SYBR Green Supermix and Opticon 2 on the CFX96 real-time RT-PCR detection system (Bio-Rad, USA). The primers used in this study are listed in Supplementary Table 1-2.

### Western blotting

Samples were lysed using an ultrasonic device in RIPA buffer containing protease and phosphatase inhibitors (Beyotime, China) and incubated on ice for 30 minutes. After centrifugation at 13,000 g for 10 minutes, the supernatant was collected, and protein concentrations were measured using the Bradford Protein Assay Kit (Sangon Biotech, China). Proteins were denatured at 100°C for 10 minutes, separated by SDS-PAGE, and transferred to NC membranes. Membranes were then blocked with 5% non-fat milk, incubated with primary antibodies overnight at 4°C, followed by secondary antibodies for 1h at room temperature. Detection was conducted, using an ECL substrate and analyzed via ImageJ software. Details of the primary and secondary antibodies used in the Western blotting experiments can be found in Supplementary Table 3.

### Detection of renal glyoxylic acid crystals by hematoxylin and eosin (HE) staining

Deposits of glyoxylic acid crystals were observed on the renal surfaces stained with HE, visualized in polarized light optical micrographs (Zeiss, Oberkochen, Germany). Photoshop software was used to splice the photographed parts of the kidney into a complete picture of the kidney.

### Chromatin immunoprecipitation (ChIP) assay

During the ChIP process, cells are first collected post-trypsin digestion and washed twice with phosphate-buffered saline (PBS). Crosslinking is then initiated by exposing the cells to formaldehyde at room temperature for 10 minutes, followed by neutralization with glycine and precipitation at 4°C. The cells are subsequently washed and prepared for nuclei isolation by successive incubations with different lysis buffers, centrifugation steps, and solubilization using a designated buffer. Sonication is employed to shear the chromatin, after which the fragments are centrifuged to remove the supernatant. Samples are then divided into immunoprecipitation (IP), IgG (control), and Input groups, to which specific antibodies are added and incubated at 4°C for 2-4h. Magnetic beads are used to collect the immunoprecipitated complexes, which are washed to remove non-specific bindings. The bound DNA is eluted by heating the samples in elution buffer at 65°C for 15 minutes, with the process repeated to ensure complete elution. Post-elution, de-crosslinking of the DNA-protein complexes is achieved by incubation at 65°C for 6h or overnight, followed by digestion with RNase A and Proteinase K at 37°C to separate the DNA from proteins. DNA is then precipitated using phenol/chloroform/isoamyl alcohol mixture, transferred to an aqueous phase, and subsequently precipitated with NaCl, glycogen, and anhydrous ethanol at -20°C. The final DNA precipitate is washed and resuspended for quantification and quality assessment using a NanoDrop spectrophotometer and agarose gel electrophoresis to determine DNA concentration and fragment size, respectively. Details in the experiments can be found in Supplementary Table 1,3 and 4.

### Immunohistochemistry (IHC)

The sections were deparaffinized in xylene (3×15 min) and ethanol series solution (5 min each), then washed in water. Antigen retrieval was performed as specified, keeping the slides moist, and then washed in PBS (3×5 min). Block endogenous peroxidase with 3% hydrogen peroxide (20 min), followed by washing in PBS (3×5 min). 3% BSA (30 min) was used for serum blocking, then the samples were incubated with primary antibody overnight at 4°C. After the samples were washed with PBS (3×5 min), HRP-secondary antibody was added and incubated (50 min). Stain with DAB, monitor under microscope, and the reaction was stopped with water. The samples were counterstain with hematoxylin, dehydrated with ethanol and xylene, and mounted with resin. Images were captured with fluorescence microscope (Olympus IX83, Japan, Zeiss Axio Observer AL, Germany).

### HE staining

The sections were deparaffinized in xylene (2×20 min), followed by ethanol (5 min each) and a tap water wash. Stained with hematoxylin for 3-5 minutes, rinsed with tap water, the sections were differentiated by bluing solution, followed by a running water rinse; Dehydrated through ethanol gradients (85% and 95% for 5 min each), then stained in eosin for 5 minutes; Dehydrated in anhydrous ethanol (3×5 min), cleared in xylene (2×5 min), and mounted with neutral resin. Images were captured and analyzed using a Nikon Eclipse E100 microscope.

### PAS staining

The samples were deparaffinized with xylene (2×20 min), ethanol (5 min each), and rinse with water; Stained with periodic acid for 15 min, rinsed, then washed twice with distilled water; Stained with Schiff's reagent for 30 min in the dark, rinsed with running water for 5 min; Stained with hematoxylin for 3-5 min, rinsed, differentiated, and rinsed again; Dehydrated with anhydrous ethanol (3×5 min), cleared with xylene (2×5 min), and mounted with neutral resin. The images were captured and analyzed using a Nikon Eclipse E100 microscope.

### MASSON staining

The samples were deparaffinized with xylene (2×20 min), ethanol (5 min each), and rinsed with water; Soaked in potassium dichromate overnight, then rinsed; Stained with iron hematoxylin for 3 min, rinsed, differentiated, and rinsed again; Stain with Ponceau Acid Fuchsin for 5-10 min, rinse, then stain with phosphomolybdic acid for 1-3 min. Directly stain with aniline blue for 3-6 min, differentiate with 1% glacial acetic acid, dehydrate, clear with xylene, and mount. Capture and analyze images using a Nikon Eclipse E100 microscope.

### Sirius Red staining

The specimens were deparaffinized with xylene (2×20 min), ethanol (5 min each), and rinsed with water. It was stained with Sirius Red solution for 8-10 min, dehydrated, cleared with xylene for 5 min, and mounted with neutral resin. Images were captured and analyzed using a Nikon Eclipse E100 microscope.

### Immunofluorescence (IF)

The cells were permeabilized with 200 μL of Saponin for 15 minutes at room temperature, followed by 3 washes (5 min each). They then were blocked with 200 μL of 3% BSA for 20 minutes, then incubated with 200 μL of diluted primary antibody (1:200) overnight at 4°C. After 3 washes, incubated with 200 μL of secondary antibody (1:400) at 37°C for 30 minutes in the dark. Following 3 washes, the cells were stained with 200 μL of DAPI for 5 minutes at room temperature in the dark. After 3 more washes, they were blotted dry and mounted with antifade medium. Images were captured using an Olympus IX83 fluorescence microscope.

### Lactate assay experiment protocol

Using the Abbkine CheKine™ Micro Lactate Assay Kit (Cat. #KTB1100), prepare standard, blank, and test sample wells. 50 μL were added to standard wells; for test wells, 40 μL sample dilution and 10 μL test sample (5x dilution) were added and mixed gently. 50 μL enzyme reagent were added to all wells except blanks and incubated at 37°C for 60 minutes. Wash wells 5 times with a 30x diluted washing solution. 50 μL Color Developer A and B were added and mixed, then incubated at 37°C in the dark for 15 minutes. 50 μL stop solution were added. The absorbance was measured at 450 nm, using the blank for calibration.

### Flow CytoLetry assay - detection of 2-NBDG uptake

Cells were seeded at a density of 4×10⁴ cells per well in a 6-well plate and incubated for 24 to 48 hours in a controlled incubator. Upon completion of the incubation, the old medium was carefully removed and replaced with fresh medium containing 10 µM of 2-NBDG. The cells were then incubated at 37°C for an additional 60 minutes. Post-incubation, the 2-NBDG-containing medium was delicately removed, and the cells were washed twice with pre-chilled PBS to halt further uptake of 2-NBDG. The cells were resuspended in fresh medium and promptly processed for flow cytometry analysis within 30 minutes, ensuring that the experimental data was accurate. This entire process required strict control over time and temperature to ensure the reliability of the experimental outcomes.

### Seahorse XF glycolysis stress test kit protocol

In the Seahorse XF Glycolysis Stress Test Kit protocol, transfected and control cells were seeded at a density of 2,000 cells per well in a 96-well plate and incubated overnight to allow proper cell attachment. Following this, the cells were washed and cultured in MEM medium that had been adjusted to exclude glucose, sodium bicarbonate, and other buffering systems. This specialized medium was incubated with the cells for 1h at 37°C within a CO_2_-free environment to prevent any external influences on the measurements. The Seahorse Extracellular Flux Analyzer (XF-96) was then employed to measure the extracellular acidification rate (ECAR). During the assay, the device sequentially injected 10 mmol/L glucose, 1 mmol/L oligomycin, and 100 µL/L 2-Deoxy-D-glucose (2-DG) into each well, while continuously recording changes in ECAR. The data analysis was performed by defining non-glycolytic acidification as the baseline ECAR before the addition of glucose, glycolysis as the ECAR post glucose injection, glycolytic capacity as the ECAR after the administration of oligomycin (which inhibits mitochondrial function), and glycolytic reserve as the difference between glycolytic capacity and glycolysis. To verify the results, the protocol included the use of 2-DG to block the cellular glycolysis pathway, which led to a decline in ECAR. This reduction in ECAR validated that the observed increases in ECAR during the test were indeed attributable to cellular glycolysis. The meticulous control of medium components and the sequential addition of specific compounds, coupled with precise ECAR measurements and data analysis, ensured the accuracy and reliability of the glycolysis stress test results.

### Biochemical indicator detection protocol

Serum samples from C57 mice were prepared. Leadman or Changchun Huili reagent kits (containing R1 and R2) were used for detecting creatinine and urea nitrogen levels. For single reagent methods, they were used directly; for double reagent methods, R1 and R2 were applied separately. Appropriate parameters were configured on a fully automated biochemical analyzer, and the samples were loaded. The analyzer was allowed to automatically perform the measurements. After detection, the results were exported in Excel format.

### Statistical analysis

All data were derived from at least three independent experiments, with values presented as mean ± SD. The statistical software used was Graphpad 8.0.1, R (4.2.0.1), SPSS 22.0, ImageJ. Initially, raw data were tested for normality and analyzed using a one-sample Kolmogorov-Smirnov nonparametric test with SPSS 22.0 software. For comparisons between two groups in animal and cellular experiments, a two-tailed unpaired Student's t-test was used. For comparisons among more than two groups, one-way ANOVA followed by Bonferroni's post-hoc test was applied. Spearman's correlation test was used to calculate the correlation coefficient. To minimize bias, all statistical analyses were conducted in a blinded manner. Statistical significance is indicated by *p < 0.05, **p < 0.01, and ***p < 0.001.

### Additional materials

The siRNA and plasmid sequence numbers can be found in Supplementary Table 4. Specific parameters and methods are provided in Supplementary Table 5 for all products used in the experiment, including COM, Gly, activators, and inhibitors.

## Result

### COM-induced damage triggered EMT, and IGF1R was positively correlated with EMT

**Figure 1:**
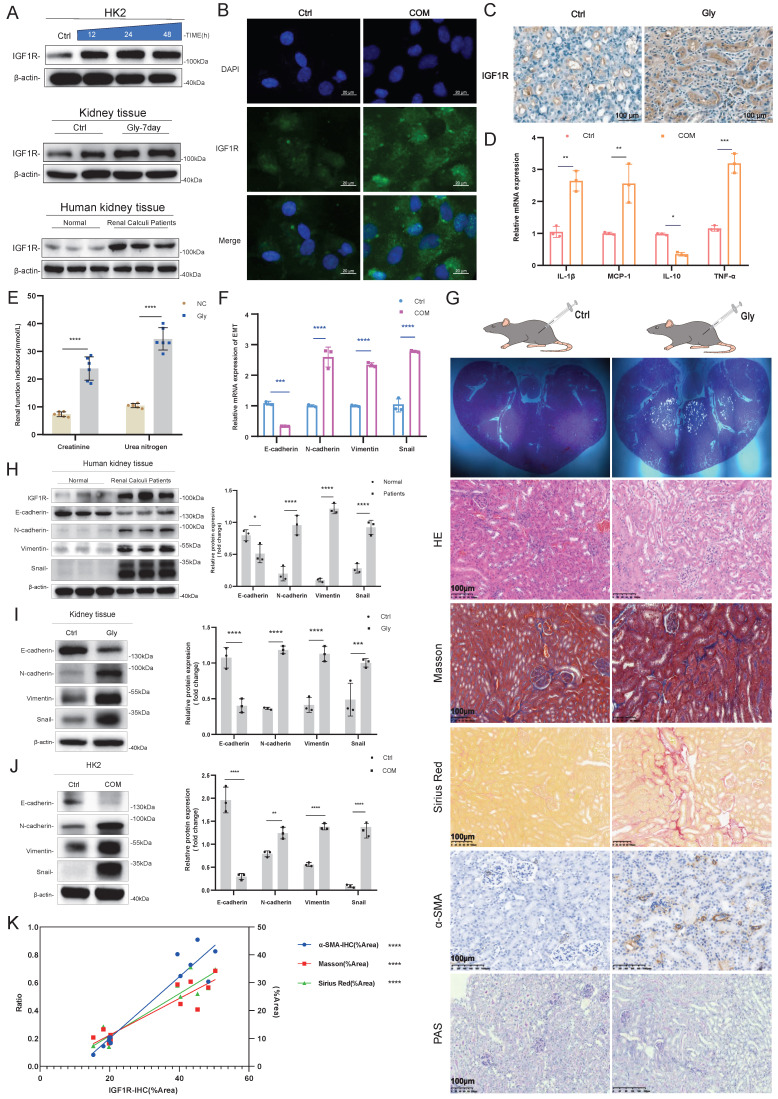
** Validation of the Correlation between EMT Induction and IGF1R in a COM Injury Model.** Legend:** (A)** Western blotting analysis of IGF1R expression in kidney tissues from patients with kidney stones, C57BL/6 mouse kidney tissues, and HK2 cells. **(B)** Immunofluorescence analysis of IGF1R expression in HK2 cells. **(C)** Immunohistochemistry analysis of IGF1R expression in C57BL/6 mice. **(D)** Quantitative PCR analysis of inflammatory markers in the *in vitro* COM model. **(E)** Assessment of changes in serum creatinine and urea nitrogen levels in mice with Gly damage. **(F)** Quantitative PCR analysis of EMT-related markers (E-cadherin, N-cadherin, Vimentin and Snail) in the *in vitro* COM model. **(G)** Polarized light microscopy to validate the extent of Gly infection in renal tissues. HE staining for comparative analysis of renal tissue structural changes. Masson staining, Sirius Red staining and α-SMA immunohistochemistry for comparative analysis of EMT changes in renal tissues. PAS staining for comparison of glycogen changes in renal tissues. **(H)** Western blotting analysis of EMT-related markers (E-cadherin, N-cadherin, Vimentin and Snail) in kidney tissues from patients with kidney stones. **(I)** Western blotting analysis of EMT-related markers (E-cadherin, N-cadherin, Vimentin and Snail) in tissues induced by Gly. **(J)** Western blotting analysis of EMT-related markers (E-cadherin, N-cadherin, Vimentin and Snail) in the *in vitro* COM model. **(K)** Correlation analysis of IGF1R quantification data with α-SMA, Sirius Red staining and Masson staining quantification data in immunohistochemistry. Note: ns: ≥0.05, *: <0.05, **: <0.01, ***: <0.001, ****: <0.0001. The data of relative protein levels are presented as fold change values to the control group of three independent experiments. Abbreviations: COM, calcium oxalate monohydrate. Gly, Glyoxylic acid.

First, our experiments revealed that renal Gly injury triggers EMT, and we explored the correlation between IGF1R and EMT in the COM. Through Western Blotting assays, IF assays and IHC experiments, we confirmed that IGF1R protein is highly expressed in the human kidney, mouse kidney tissue, and HK2 cells (Figure 1A, B, and C). Additionally, inflammation (IL-1β, MCP-1, IL-10 and TNF-α) increased, and renal function indicators, such as creatinine and urea nitrogen, were significantly elevated. At the mRNA level, we found that COM injury led to upregulation of N-cadherin, Vimentin and Snail that are EMT-related markers (Figure 1D-F). We further performed HE staining, Masson staining, PAS staining, Sirius Red staining, and α-SAM immunohistochemistry on kidney tissues to demonstrate that Gly injury in the kidney had triggered EMT changes (Figure 1G). Furthermore, we validated the significant trends of EMT markers through Western Blotting assays in human, mouse tissues, and HK2 cell (Figure 1H-J), with N-cadherin, Vimentin and Snail upregulated, and E-cadherin downregulated. Finally, we quantified the IHC data of IGF1R, as well as the data from Masson staining, PAS staining, Sirius Red staining, and α-SAM immunohistochemistry, and performed statistical analysis, which revealed a significant correlation between IGF1R and EMT (Figure 1K).

### Inhibition of IGF1R helps alleviate renal function deterioration and the EMT trend

**Figure 2:**
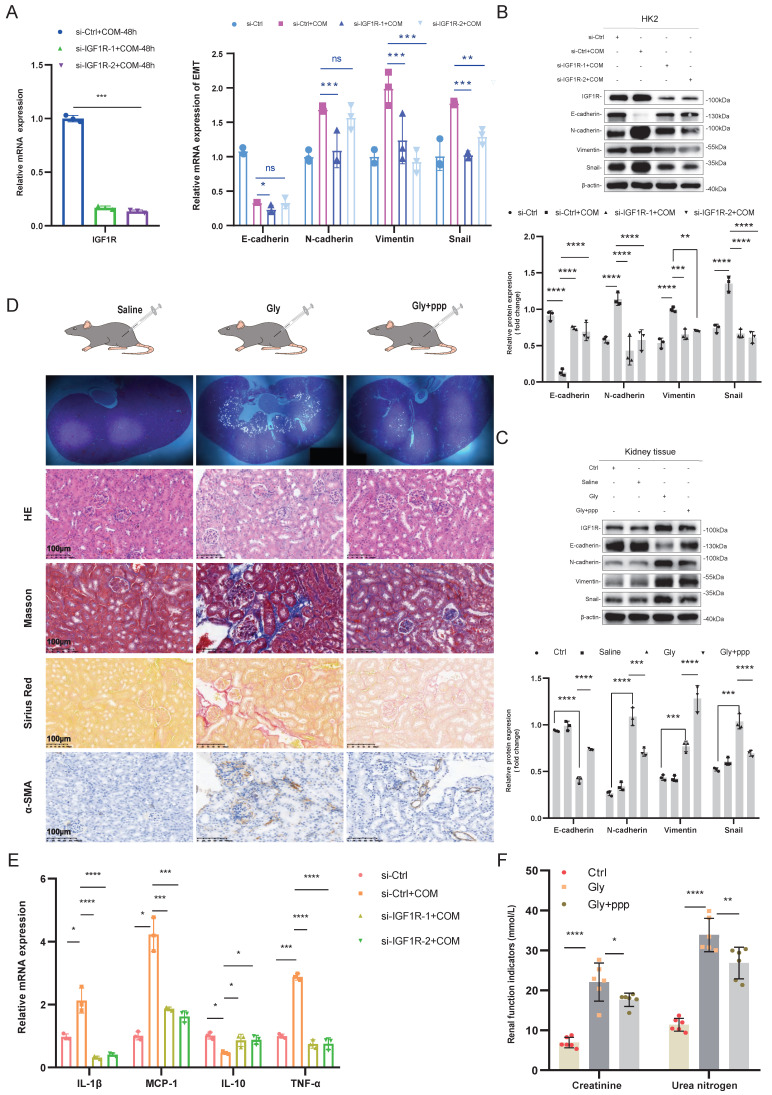
** Inhibition of IGF1R Improves Renal Function and EMT Progression.** Legend:**(A)** Verification of si-IGF1R knockdown efficiency and quantitative PCR analysis of EMT-related markers (E-cadherin, N-cadherin, Vimentin and Snail) following IGF1R knockdown *in vitro*. **(B)** Western blotting analysis of EMT-related markers (E-cadherin, N-cadherin, Vimentin and Snail) following IGF1R knockdown *in vitro*. **(C)** Western blotting analysis of EMT-related markers (E-cadherin, N-cadherin, Vimentin and Snail) following IGF1R inhibition *in vivo*. **(D)** Polarized light microscopy to validate the extent of Gly infection in renal tissues following IGF1R inhibition. Comparison of EMT changes in renal tissues treated with IGF1R inhibitor (PPP) using HE staining, Masson staining, Sirius Red staining, and α-SMA immunohistochemistry. **(E)** Quantitative PCR analysis of changes in inflammation-related markers following IGF1R knockdown *in vitro*. **(F)** Measurement of serum creatinine and urea nitrogen level. Note: The data of relative protein levels are presented as fold change values to the control group of three independent experiments. ns: ≥0.05, *: <0.05, **: <0.01, ***: <0.001, ****: <0.0001. Abbreviations: COM, calcium oxalate monohydrate. Gly, Glyoxylic acid.

After confirming the significant correlation between IGF1R and EMT, we further explored the biological function of IGF1R. *In vitro* experiments showed that IGF1R knockdown by using siRNA transfection inhibited the expression of EMT-related markers at both mRNA and protein levels compared to that of si-Ctrl+COM group. Specifically, E-cadherin expression was enhanced, while N-cadherin, Vimentin, and Snail expressions were reduced, and inflammation markers were also decreased (Figure 2A, B, and E). The function of IGF1R was also validated at the tissue level. Compared to the Gly group, the PPP+Gly group showed more intact tubular epithelial cells in HE staining, and the degree of fibrosis was reduced as indicated by Masson staining, Sirius Red staining, and α-SAM immunohistochemistry (Figure 2C). At the protein level, Western Blotting results demonstrated a decrease in the EMT trend (Figure 2D), and renal function indicators showed partial recovery after the addition of PPP (Figure 2F).

### IGF1R promotes COM induced EMT progression through JAK2/STAT3 pathway

**Figure 3:**
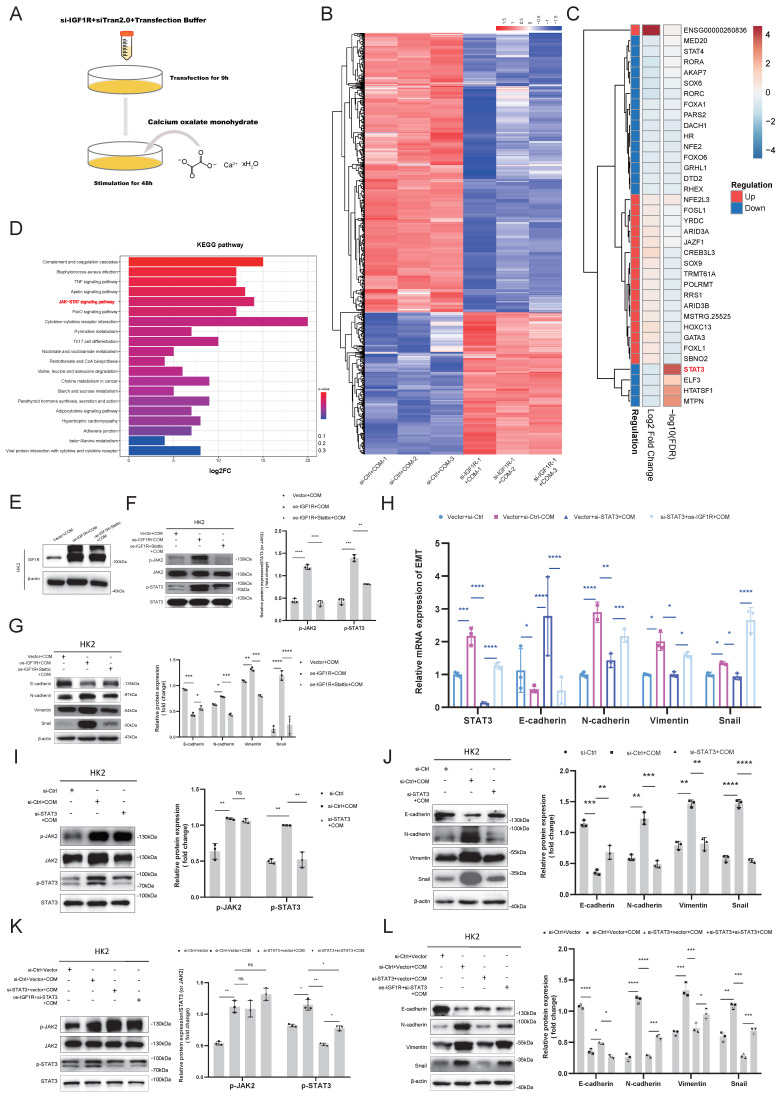
** IGF1R Mitigates COM-Induced EMT through the JAK2/STAT3 Pathway.** Legend:**(A)** Flowchart of the process for transfecting HK2 cells with si-IGF1R and establishing the COM model prior to transcriptome sequencing. **(B)** Heatmap showing differential gene expression between the COM group and the si-IGF1R+COM group based on transcriptome sequencing data. **(C)** Heatmap of false discovery rate (FDR) values for all differentially expressed transcription factors between the COM group and the si-IGF1R+COM group based on transcriptome sequencing data. **(D)** KEGG enrichment pathway analysis of transcriptome sequencing data comparing the COM group with the si-IGF1R+COM group. **(E)** Western blotting analysis confirmed the efficiency of IGF1R overexpression following plasmid transfection. **(F)** Western blotting analysis assessed the effects of oe-IGF1R and Stattic on the JAK2/STAT3 signaling pathway. **(G)** Western blotting analysis validated the impact of the IGF1R-JAK2/STAT3 axis on EMT-related markers (E-cadherin, N-cadherin, Vimentin and Snail) *in vitro*. **(H)** Quantitative PCR analysis of the effect of IGF1R on downstream JAK2/STAT3 signaling pathway changes and its impact on EMT-related markers (E-cadherin, N-cadherin, Vimentin and Snail). **(I)** Western blotting analysis of the effect of STAT3 knockdown on the JAK2/STAT3 signaling pathway *in vitro*. **(J)** Western blotting analysis of the impact of STAT3 knockdown on EMT-related markers (E-cadherin, N-cadherin, Vimentin and Snail). **(K)** Western blotting analysis of the effect of IGF1R on the JAK2/STAT3 pathway *in vitro*. **(L)** Western blotting analysis of the relationship between the IGF1R-JAK2/STAT3 axis and changes in EMT-related markers (E-cadherin, N-cadherin, Vimentin and Snail). Note: The data of relative protein levels are presented as fold change values to the control group of three independent experiments. Abbreviation: COM, Calcium Oxalate Monohydrate; FDR, false discovery rate. ns: ≥0.05, *: <0.05, **: <0.01, ***: <0.001, ****: <0.0001.

We further explored the mechanisms of IGF1R in COM injury induced EMT. We performed transfection of IGF1R siRNA or negative control siRNA in HK2 cells with COM treatment as shown in Figure 3A, followed by RNA-seq analysis. The sequencing results indicated that there were 720 differentially expressed genes between the COM group and the si-IGF1R+COM group, with 242 genes upregulated and 478 genes downregulated. By intersecting the human transcription factor collection with the differentially expressed genes, we screened and identified all differentially expressed transcription factors, and STAT3 showed the strongest correlation with IGF1R. STAT3 exhibited strong signals in both differential expression and KEGG enrichment pathways (Figure 3B-D). To validate the role of the IGF1R and JAK2/STAT3 axis, we first assessed the efficiency of IGF1R overexpression in the COM model, as shown in Figure 3E. Subsequently, as illustrated in Figure 3F, successful overexpression of IGF1R led to an enhancement of the JAK2/STAT3 pathway, which was attenuated by STAT3 inhibitor Stattic. Following the demonstration that IGF1R positively regulates the JAK2/STAT3 pathway, as shown in Figure 3G that, in the COM-induced injury model, the overexpression of IGF1R (oe-IGF1R+COM group) resulted in elevated levels of EMT-related markers, including E-cadherin, N-cadherin, Vimentin and Snail. Treatment with Stattic led to a decrease in JAK2/STAT3 pathway activation (phosphorylation), which mitigated the EMT progression (Figure 3G). Furthermore, siRNA knockdown of STAT3 led to a corresponding reversal of EMT-related markers at the mRNA level (E-cadherin, N-cadherin, Vimentin and Snail), suggesting that EMT is also regulated by STAT3 at the mRNA level (Figure 3H). At the protein level, STAT3 gene knockdown also inhibited the EMT progression induced by COM (Figures 3I-J). Finally, rescue experiments demonstrated that IGF1R promotes EMT progression through the STAT3/JAK2 pathway (Figures 3K-L).

### IGF1R activates the glycolytic pathway induced by COM

**Figure 4:**
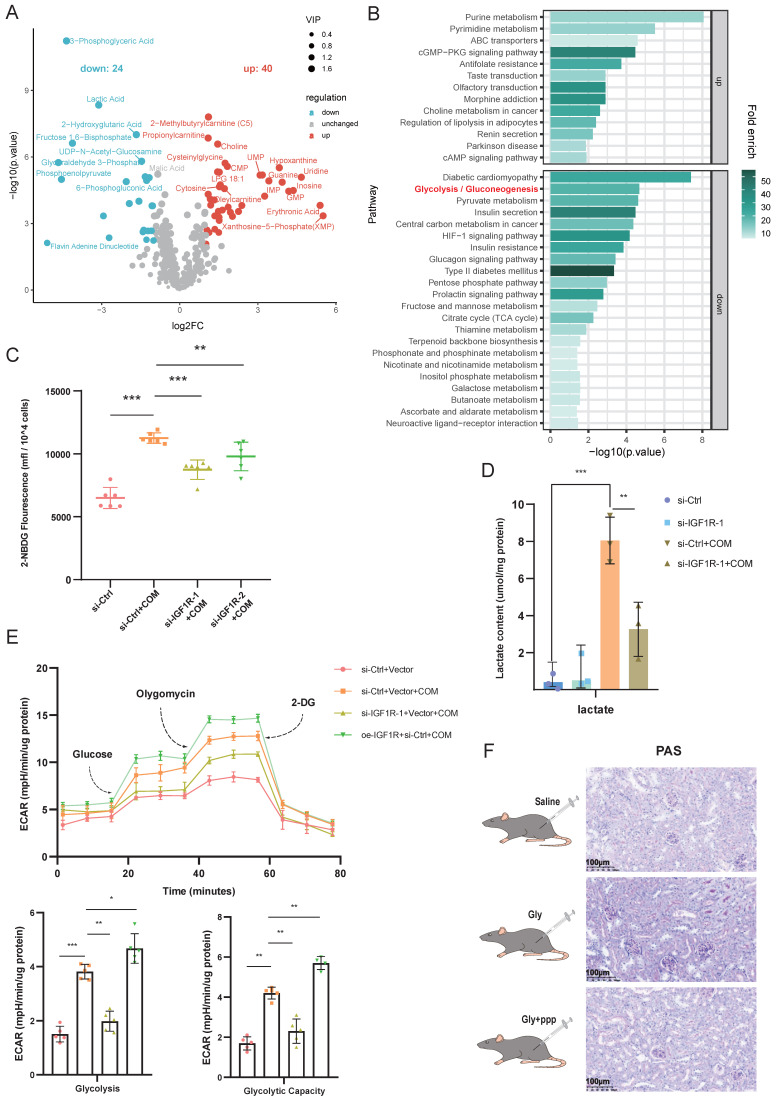
** IGF1R Regulates Glycolytic Metabolism Induced by COM.** Legend: **(A)** Volcano plot of untargeted metabolomics sequencing results showing differential metabolites between the COM group and the si-IGF1R+COM group. **(B)** Heatmap of KEGG enrichment pathways from untargeted metabolomics sequencing data comparing the COM group with the si-IGF1R+COM group. **(C)** Flow cytometry vitality assay detecting changes in glucose uptake levels in HK2 cells under different treatments involving IGF1R. **(D)** Measurement of lactate metabolism levels in HK2 cells under different treatments involving IGF1R. **(E)** Seahorse assay showing changes in glycolytic capacity of HK2 cells under different treatments involving IGF1R. **(F)** PAS staining analysis of glycogen levels in kidney tissue after inhibition of IGF1R (PPP). Note: Abbreviation: COM, Calcium Oxalate Monohydrate. ECAR, extracellular acidification rate. 2-DG, 2-Deoxy-D-glucose. PPP, Picropodophyllotoxin. ns: ≥0.05, *: <0.05, **: <0.01, ***: <0.001, ****: <0.0001.

Given that multiple studies have reported the involvement of IGF1R in cellular metabolic functions^23^, ^24^, we conducted untargeted metabolomics sequencing on HK2 cells with the same treatment (COM group vs. COM+si-IGF1R group). The heatmap results from the sequencing showed that as IGF1R expression decreased, many glycolytic products, such as pyruvate, lactate, fructose-6-phosphate, and fructose-1,6-bisphosphate, also decreased (Figure 4A). Glycolysis was also enriched in pathway enrichment analysis (Figure 4B). Thus, we conducted a series of experiments focusing on the impact of IGF1R on cellular functions related to glycolysis. First, we validated the data obtained. In terms of glucose uptake by the cells, the COM group showed an increased ability to take up glucose, which is inhibited by IGF1R knockdown with COM treatment (Figure 4C).

And, it was found that the lactate content was elevated in the COM group, while IGF1R knockdown significantly reduced the lactate content caused by COM (Figure 4D). As indicated by the untargeted metabolomics results, in the Seahorse assay, the ECAR of the COM group was increased, while it was decreased in the COM+si-IGF1R group, which was also validated in terms of glycolysis and glycolytic capacity (Figure 4E). *In the in vivo* experiments, PAS staining was performed on kidney tissues, and the results showed that glycogen levels decreased when the biological function of IGF1R was inhibited (Figure 4F). These results demonstrate that COM (or Gly) significantly affects glycolysis function, and IGF1R plays a crucial role in this progression.

### IGF1R-STAT3 pathway promotes glycolysis metabolism by transcriptionally upregulating LDHA

**Figure 5:**
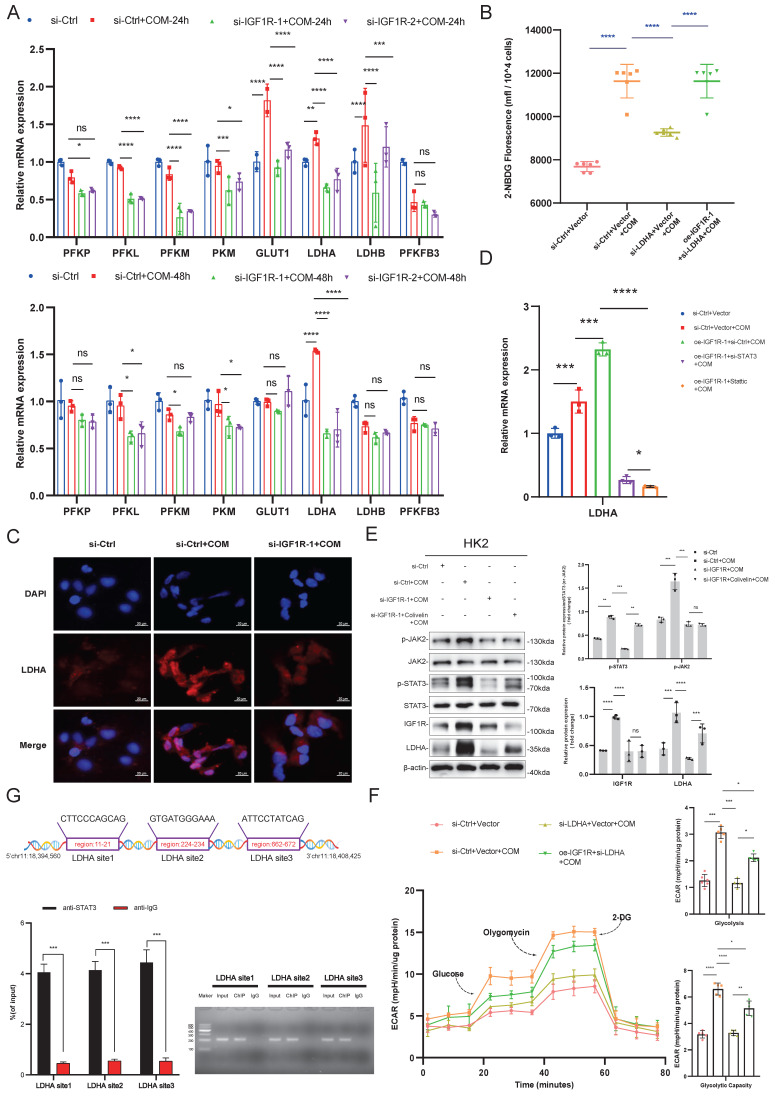
** IGF1R-JAK2/STAT3 Pathway Regulates Glycolytic Metabolism by Transcriptionally Modulating LDHA.** Legend:**(A)** Quantitative PCR analysis of changes in key glycolytic enzyme proteins after IGF1R knockdown at 24 and 48h of COM stimulation. **(B)** Flow cytometry vitality assay measuring changes in glucose uptake levels in cells after knockdown of LDHA expression. **(C)** Immunofluorescence assay verifying the effect of IGF1R on LDHA *in vitro*. **(D)** Quantitative PCR analysis of the effect of the IGF1R-STAT3 pathway on LDHA *in vitro*. **(E)** Western blotting analysis confirming the impact of the IGF1R-STAT3 pathway on LDHA. **(F)** Seahorse assay assessing the effect of the IGF1R-LDHA pathway on glycolytic activity in HK2 cells. **(G)** Chromatin immunoprecipitation assay confirming strong binding sites of STAT3 to the LDHA gene. Note: The data of relative protein levels are presented as fold change values to the control group of three independent experiments. Abbreviation: COM, Calcium Oxalate Monohydrate. ECAR, extracellular acidification rate. 2-DG, 2-Deoxy-D-glucose. LDHA, lactate dehydrogenase A. ns: ≥0.05, *: <0.05, **: <0.01, ***: <0.001, ****: <0.0001. p-STAT3#: Data obtained by comparing the gray value of STAT3

After confirming that IGF1R is involved in the glycolytic process induced by COM, we further explored which key enzymes in glycolysis are associated with IGF1R. We tested the association of IGF1R with key enzymes in glycolysis and observed their changes. As shown in Figure 5A, with the induction of COM, GLUT1, LDHA, and LDHB were all upreguated after 24h, but after 48h, only LDHA remained significantly upregulated. After knocking down IGF1R for 48h, the decrease in LDHA was most pronounced. Based on those phenomena, we speculated that LDHA is significantly correlated with IGF1R and plays a vital role in the EMT progression. Through rescue experiments at both the mRNA and protein levels, it was confirmed that IGF1R may regulate the expression of LDHA through STAT3 activation. As shown in Figure 5B, LDHA knockdown decreased the glucose uptake capacity in HK2 cells, while in the oe-IGF1R+si-LDHA+COM group, glucose uptake was recovered. The immunofluorescence experiments in Figure 5C showed that LDHA expression decreased with IGF1R knockdown. Furthermore, it was verified at both mRNA and protein levels that IGF1R upregulates LDHA expression through the JAK2/STAT3 pathway (Figure 5D-E). In the glycolysis stress test shown in Figure 5F, glycolytic capacity decreased in HK2 cells with LDHA knockdown, while in the oe-IGF1R+si-LDHA+COM group, glycolysis was partially recovered. This was also validated in terms of glycolytic and glycolytic capacity. The above results indicate that IGF1R may upregulate LDHA and influence glycolysis. Additionally, it was confirmed by the ChIP experiments that STAT3 has strong binding sites in the promoter of LDHA gene (Figure 5G). These results suggest that IGF1R may induce metabolic reprogramming in HK2 cells through upregulating JAK2/STAT3-LDHA signaling.

### IGF1R enhances EMT progression through glycolysis

**Figure 6:**
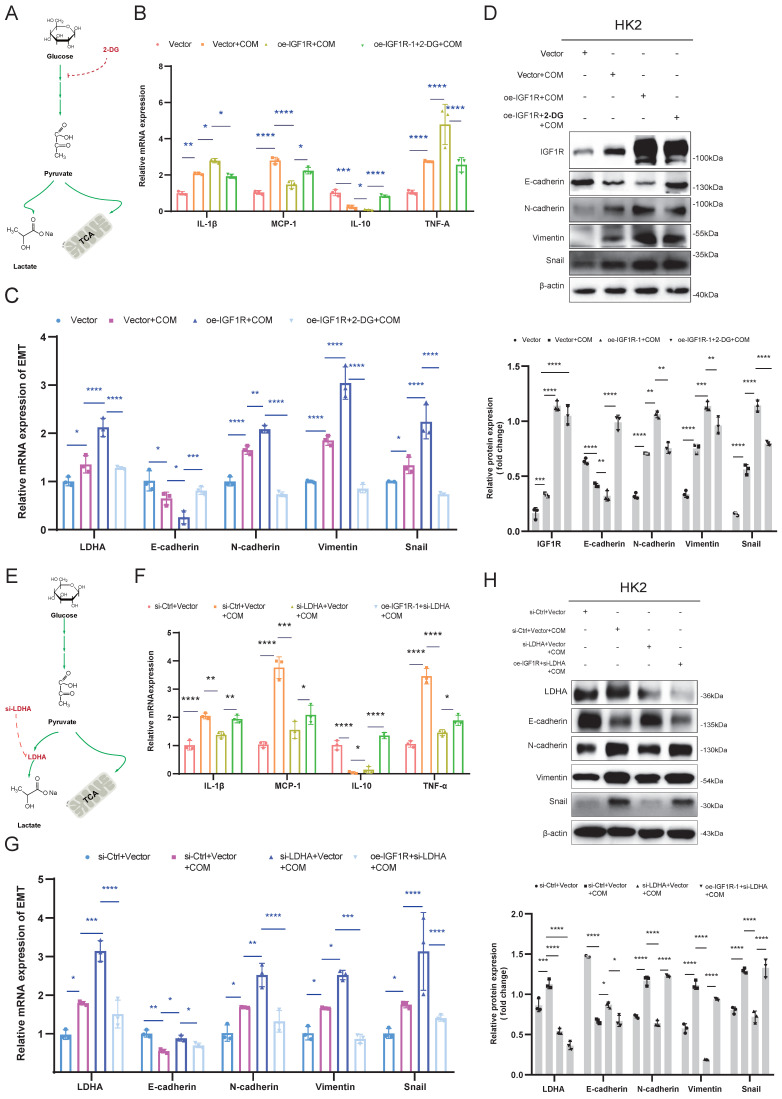
** IGF1R Influences EMT progression Through Glycolysis.** Legend: **(A)** Schematic diagram of 2-DG blocking glycolysis. **(B)** Quantitative PCR analysis of the effect of IGF1R function on inflammation-related markers through inhibition of the glycolysis pathway. **(C)** Quantitative PCR analysis of the effect of IGF1R function on EMT-related markers (E-cadherin, N-cadherin, Vimentin and Snail) through inhibition of the glycolysis pathway. **(D)** Western blotting analysis of the effect of IGF1R function on EMT-related markers (E-cadherin, N-cadherin, Vimentin and Snail) through inhibition of the glycolysis pathway. **(E)** Schematic diagram of LDHA knockdown affecting the anaerobic glycolysis pathway. **(F)** Quantitative PCR analysis of the effect of IGF1R function on inflammation-related markers through inhibition of LDHA. **(G)** Quantitative PCR analysis of the effect of IGF1R function on EMT-related markers (E-cadherin, N-cadherin, Vimentin and Snail) through inhibition of LDHA. **(H)**Western blotting analysis of the effect of IGF1R function on EMT-related markers (E-cadherin, N-cadherin, Vimentin and Snail) through inhibition of LDHA. Note: The data of relative protein levels are presented as fold change values to the control group of three independent experiments. Abbreviation: COM, Calcium Oxalate Monohydrate. 2-DG, 2-Deoxy-D-glucose. LDHA, lactate dehydrogenase A. ns: ≥0.05, *: <0.05, **: <0.01, ***: <0.001, ****: <0.0001

After confirming that IGF1R promotes glycolysis by regulating LDHA, we further explored whether IGF1R affects the EMT progression caused by COM injury through glycolysis. On the basis of COM-induced injury, the HK2 cells were treated with 2-DG to inhibit glycolysis, and it was observed that EMT-related markers were alleviated at both mRNA and protein levels, along with a reduction in inflammation markers (Figure 6B-D). Furthermore, LDHA knockdown decreased the EMT progression and inflammation markers at both mRNA and protein levels (Figure 6F-G). From the above results, it can be concluded that IGF1R enhanced the EMT progression by upregulating LDHA expression in the glycolysis through the JAK2/STAT3 signaling.

### IGF1R enhances glyoxylic acid crystallization-induced EMT via JAK2/STAT3-LDHA axis

**Figure 7:**
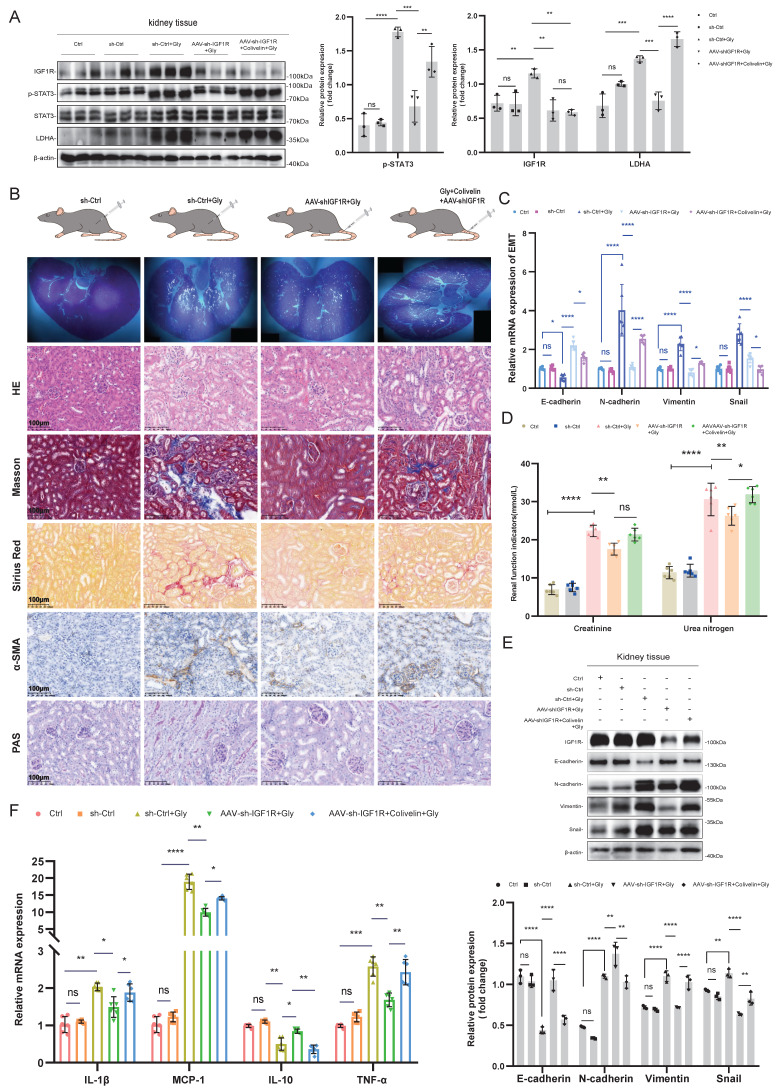
***In vivo* validation of the IGF1R-STAT3-LDHA axis for glyoxylic acid crystallization-induced EMT.** Legend: **(A)** Western blotting analysis validating the efficiency of IGF1R knockdown and STAT3 activation *in vivo*, as well as their effects on downstream LDHA. **(B)** Polarized light microscopy used to assess the extent of renal tissue damage following Gly treatment after different processing. Comparison of renal tissue EMT and glycogen changes among different treatment groups using HE staining, Masson staining, Sirius Red staining, α-SMA immunohistochemistry, and PAS assay. **(C)** Quantitative PCR analysis investigating the progression of EMT-related markers (E-cadherin, N-cadherin, Vimentin and Snail) in the kidneys of different treatment groups *in vivo*. **(D)** Examination of the trends in creatinine and urea nitrogen levels in the kidneys of different treatment groups *in vivo*. **(E)** Western blotting analysis exploring the progression of EMT-related markers (E-cadherin, N-cadherin, Vimentin and Snail) in the kidneys of different treatment groups *in vivo*. **(F)** Quantitative PCR analysis investigating the trends of inflammation-related markers in the kidneys of different treatment groups *in vivo*. Note: The data of relative protein levels are presented as fold change values to the control group of three independent experiments. ns: ≥0.05, *: <0.05, **: <0.01, ***: <0.001, ****: <0.0001

In the *in vivo* experiments, we investigated the impact of the IGF1R-STAT3-LDHA axis on EMT induced by Gly injury using IGF1R knockdown and Colivelin, an activator of the STAT3 pathway. First, we validated the effects of AAV-shIGF1R and Colivelin on the Gly injury mouse model *in vivo*. As shown in Figure 7A, IGF1R protein upregulation and STAT3 activation (p-STAT3) were successfully verified, respectively. Further analysis including HE staining, Masson staining, PAS staining, Sirius Red staining, and α-SMA immunohistochemistry revealed that the EMT results were aligned with that in the *in vitro* experiments: IGF1R knockdown alleviated the EMT progression and decreased the glycogen content compared to the sh-Ctrl+Gly group. However, activating STAT3 on the basis of reduced IGF1R expression resulted in an increased EMT trend and glycogen content (Figure 7B). From the validation results at mRNA and protein levels in kidney tissues, AAV-shIGF1R treatment alleviated EMT, while the addition of Colivelin led to a reversal, that is enhanced the EMT trend. Inflammatory markers also responded consistently with the* in vitro* experiments (Figure 7C, E, and F). Unfortunately, the renal function (creatinine and urea nitrogen) did not show a significient reversible increase with STAT3 activation (Figure 7D). These results indicate that, IGF1R also enhances the EMT progression induced by Gly through the JAK2/STAT3-LDHA signaling* in vivo*.

## Discussion

**Figure 8:**
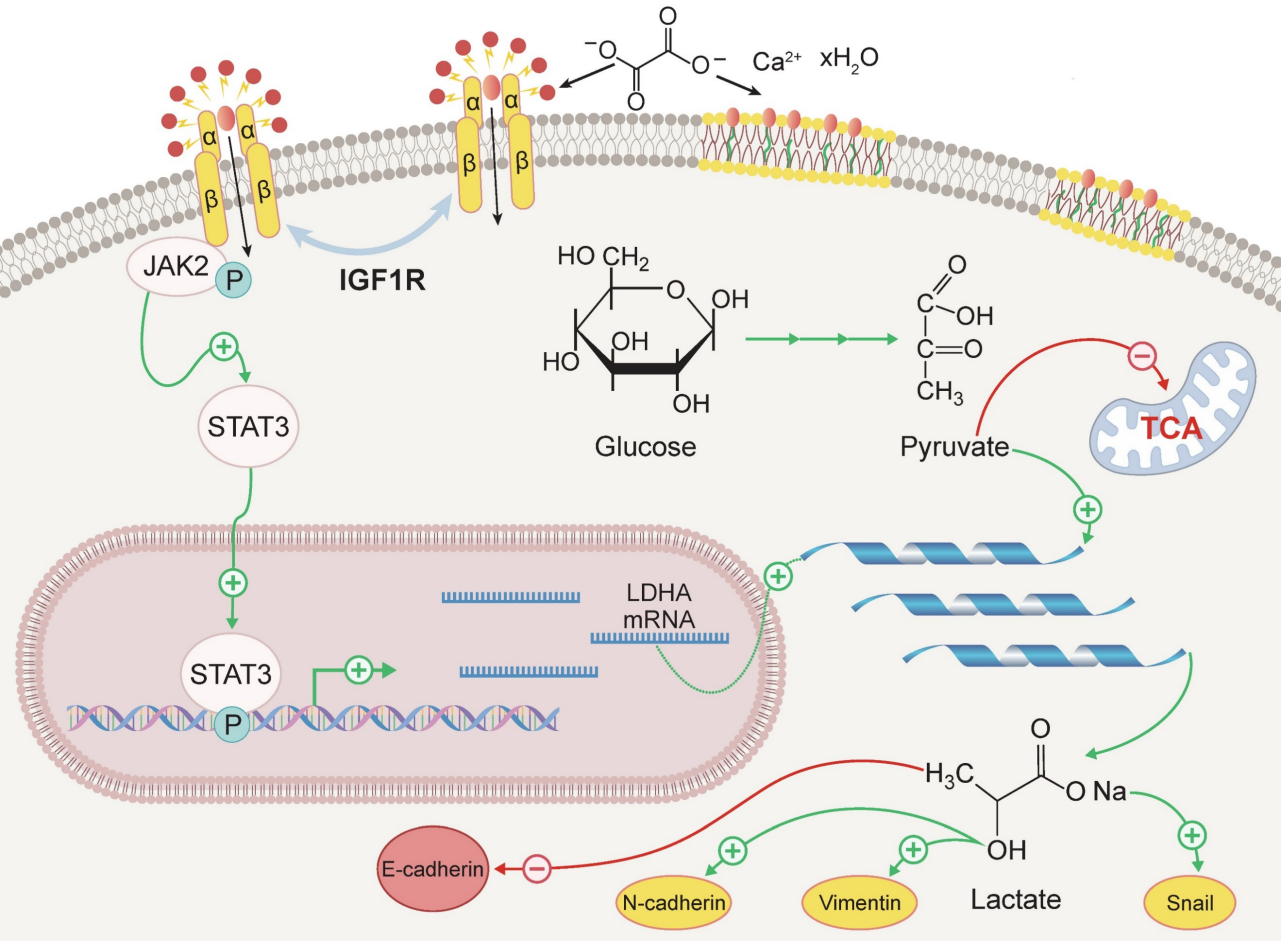
** Schematic representation of the mechanism by which IGF1R enhances EMT induced by COM.** Abbreviation: EMT, epithelial-mesenchymal transition; COM, calcium oxalate monohydrate.

The aim of this study was to investigate the role of IGF1R in COM-induced injury and its underlying mechanisms. Our key findings include: (1) IGF1R expression is elevated in COM injury models and is positively correlated with EMT; (2) Inhibition of IGF1R function alleviates renal deterioration and suppresses EMT progression; (3) IGF1R upregulates LDHA via the JAK2/STAT3 pathway, influencing glycolytic metabolism and subsequently promoting EMT progression. These discoveries offer new insights into the mechanisms of kidney stone-induced damage and provide a theoretical basis for potential therapeutic strategies. The specific biological mechanisms by which IGF1R participates, under COM induction, are illustrated in Figure 8.

Inflammatory responses play a crucial role in triggering EMT. Our study confirmed that COM elevates inflammatory cytokine levels, which is consistent with the previous reports. Cytokines such as TNF-α, IL-1β, and MCP-1 can induce EMT through various pathways, including the activation of signaling cascades (e.g., NF-κB, MAPK, PI3K/AKT), modulation of transcription factors (e.g., Snail, ZEB, TWIST), and regulation of extracellular matrix remodeling^25^-^27^. We further demonstrated the critical role of IGF1R in this process, as IGF1R inhibition significantly reduced inflammatory cytokine levels and mitigated EMT progression. This finding aligns with recent reports on IGF1R's role in inflammation and fibrosis in other organs^28^, ^29^, suggesting IGF1R as a potential molecular link between inflammation and EMT.

After confirming that IGF1R can influence the inflammation and EMT induced by COM, we considered to investigate the specific mechanisms by transcriptomic sequencing through which IGF1R links with downstream transcription factors affecting the EMT progression. As shown in Figure 3A-D, the transcriptomic data results revealed a significant impact on the STAT3 transcription factor. The mechanisms by which IGF1R influences STAT3 phosphorylation have also been studied. IGF1R can promote STAT3 phosphorylation through multiple pathways, including direct binding and phosphorylation of STAT3^30^, activation of JAK1/JAK2, PI3K/AKT or MAPK pathways can enhance STAT3 phosphorylation^31^. In our current experiment, we demonstrated that IGF1R activation under COM induction initiates the JAK2/STAT3 pathway, thereby altering the EMT trajectory.

Metabolic reprogramming is a hallmark of EMT. Enhanced glycolysis often accompanies EMT in various cancers and renal tubular epithelial cells^32^, ^33^. IGF1R has been shown to promote glycolysis in cancers^34^, ^35^, though its impact on glycolysis in the context of stone-induced injury has not been previously reported. Through a combination of untargeted metabolomic analysis and targeted experimental validation, we discovered that IGF1R upregulates LDHA via the JAK2/STAT3 pathway. This upregulation promotes glycolysis and facilitates the EMT progression. Given the well-established role of IGF1R in influencing STAT3 phosphorylation, this study focused on the regulation of downstream LDHA by JAK2/STAT3 signaling axis and its role in kidney stone formation. Previous studies have used dual luciferase reporter gene assays to demonstrate a direct interaction between STAT3 and LDHA promoter^36^, Based on this, we conducted ChIP experiments to further confirm the strong binding interaction between STAT3 and the promoter of the LDHA gene. This complex interaction network allows the IGF1R-STAT3 axis to play a critical role in various physiological and pathological processes, including the EMT associated with kidney stones observed in this study.

This novel finding highlights the intrinsic connection between metabolic reprogramming and EMT. Importantly, we are the first to reveal the role of the IGF1R-JAK2/STAT3-LDHA axis in regulating glycolysis and EMT in a kidney stone model, offering a fresh perspective on the metabolic mechanisms underlying kidney stone injury.

As a multifunctional receptor tyrosine kinase, IGF1R exerts its effects through various downstream signaling pathways^37^. In addition to the JAK2/STAT3 pathway highlighted in this study, IGF1R can also activate the PI3K/AKT and MAPK pathways, affecting the activity of EMT-related transcription factors^38^. Furthermore, IGF1R contributes to extracellular matrix remodeling and cytoskeletal reorganization, processes integral to EMT^39^. Therefore, IGF1R likely promotes EMT through multiple synergistic mechanisms.

Although direct studies on IGF1R in kidney stone-related EMT are scarce, previous researches have emphasized the significance of EMT in renal diseases. Xu J *et al.* discussed the role of TGF-β-induced EMT in renal fibrosis, which provides context for understanding the importance of EMT in kidney disorders^40^. Michael Zeisberg *et al.* explored mechanisms of tubular interstitial fibrosis, including the role of EMT, which may be relevant to tissue damage and repair following stone formation^41^, ^42^. Future research should investigate IGF1R's interactions with other signaling pathways to fully comprehend its role in the pathophysiology of nephrolithiasis.

This study also found that IGF1R silencing can improve renal function parameters, such as reducing serum creatinine and blood urea nitrogen levels. In contrast to previous research, which primarily focused on the protective effects of IGF1 and growth hormone-mediated IGF1R activation in models of other renal diseases^43^, this study specifically explored the biological functions of IGF1R. However, it is important to note that IGF1R plays a crucial role in renal development and repair^44^. Therefore, when considering IGF1R as a therapeutic target, its potential side effects and long-term implications must be carefully evaluated.

Additionally, this study validated the role of the IGF1R-JAK2/STAT3-LDHA axis in both *in vivo* and *in vitro* models, enhancing the reliability and clinical relevance of the findings. However, several limitations remain. First, we primarily focused on the impact of IGF1R on EMT, but IGF1R may also be involved in other aspects of kidney stone formation, such as calcium-phosphate metabolism[45]and oxidative stress^46^, which require further investigation in the context of kidney stone injury. Second, although we observed changes in IGF1R expression in human samples, the sample size was relatively small, and larger-scale clinical studies are needed to further validate the role of IGF1R in human nephrolithiasis.

## Conclusion

In summary, this study elucidates the critical role of IGF1R in COM-induced injury, where it regulates glycolytic metabolism through the JAK2/STAT3-LDHA axis to enhance the EMT progression. This discovery not only deepens our understanding of the mechanisms underlying kidney stone injury but also provides potential targets for developing new therapeutic strategies. Future research could further explore other molecular mechanisms regulated by IGF1R to develop more precise and effective treatments.

## Supplementary Material

Supplementary figures and tables.
