# Supplementary material for: IGF1R Enhances Calcium Oxalate Monohydrate-Induced Epithelial-Mesenchymal Transition by Reprogramming Metabolism via the JAK2/STAT3 Signaling
Source: Int J Biol Sci. 2025 Jan 1;21(1):415–32. doi: 10.7150/ijbs.104311 (PMC11667822; doi:10.7150/ijbs.104311)
Supplement: Supplementary file 1 — Supplementary figures and tables. [file ijbsv21p0415s1.pdf]

IGF1R Enhances Calcium Oxalate Monohydrate-Induced Epithelial-Mesenchymal Transition by Reprogramming Metabolism via the JAK2/STAT3 Pathway

Jiashan Pan[1,2,3*], Yi Zhang[1,2,3*], Rui Yao[1,2,3*], Mo Yang[1,2,3], Xike Mao[1,2,3], Zhenyu Song[1,2,3], Yuexian Xu[1,2,3], Yang Chen[1,2,3], Bingbing Hou[1,2,3], Xiaoying Liu[4#], Wei Wang[1,2,3#], Zongyao Hao[1,2,3#]

[1]*Department of Urology, The First Affiliated Hospital of Anhui Medical University, Hefei, China*

[2]*Institute of Urology, Anhui Medical University, Hefei, China*

[3]*Anhui Province Key Laboratory of Genitourinary Diseases, Anhui Medical University, Hefei, China*

[4]*School of Life Sciences, Anhui Medical University, Hefei 230032, China; Henan International Joint Laboratory of Non-Coding RNA and Metabolism in Cancer, Henan Provincial Key Laboratory of Long Non-Coding RNA and Cancer Metabolism, Translational Research Institute of Henan Provincial People's Hospital and People's Hospital of Zhengzhou University, Henan 450053, China.*

**These authors contributed equally to this work.*

[#]*Corresponding Author: Zongyao Hao (ORCID https://orcid.org/0009-0002-1586-5093)*

Department of Urology, The First Affiliated Hospital of Anhui Medical University and Institute of Urology and Anhui Province Key Laboratory of Genitourinary Diseases, Anhui Medical University, Jixi Road 218th, Shushan District, Hefei, Anhui, 230022, People's

Republic of China

Email: haozongyao@ahmu.edu.cn

Running title: *IGF1R Promotes EMT in COM-Induced Injury via JAK2/STAT3*

Figure number: 8

Supplemental figure number: 3

Supplemental table number: 5

Figure S1 The trend of IGF2R changes in HK2 cells induced by calcium oxalate monohydrate injury. (A) There is no significant difference in IGF2R between the COM group and the NC group at the mRNA level. (B) There is no significant difference in IGF2R between the COM group and the NC group at the protein level. COM: calcium oxalate monohydrate.

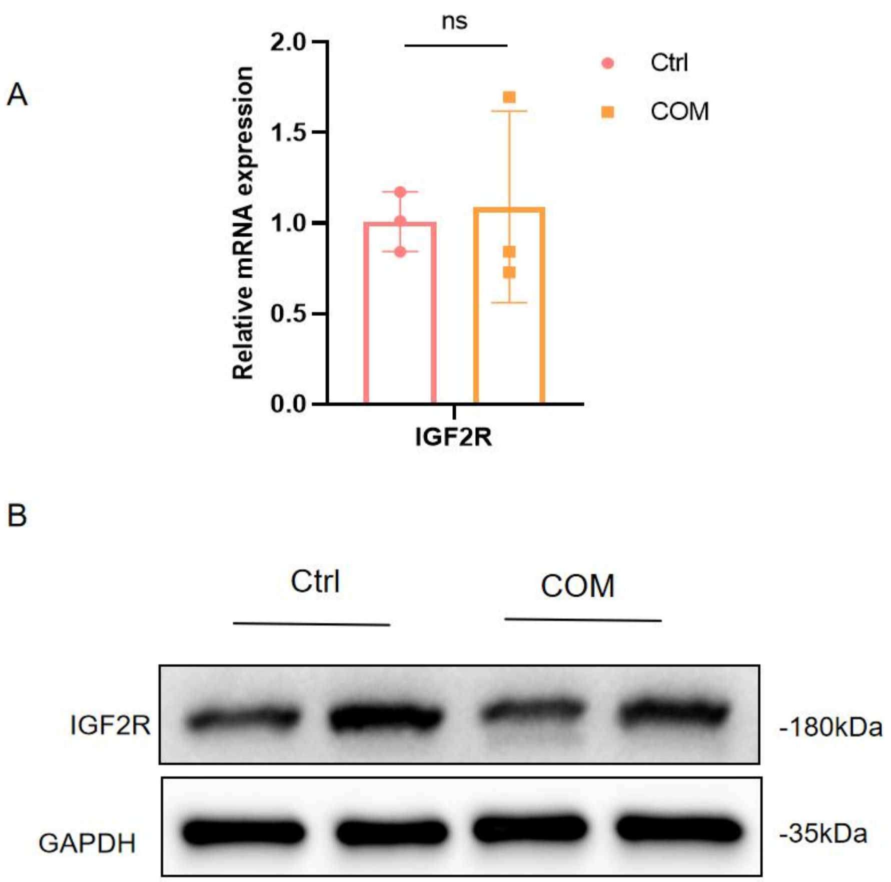

Figure S2 The results of quality control for transcriptomics and non-targeted metabolomics.

(A) PCA results of transcriptomics between the si-COM group and the si-IGF1R+COM group.

(B) PCA results of non-targeted metabolomics between the si-COM group and the si-IGF1R+COM group. COM: calcium oxalate monohydrate.

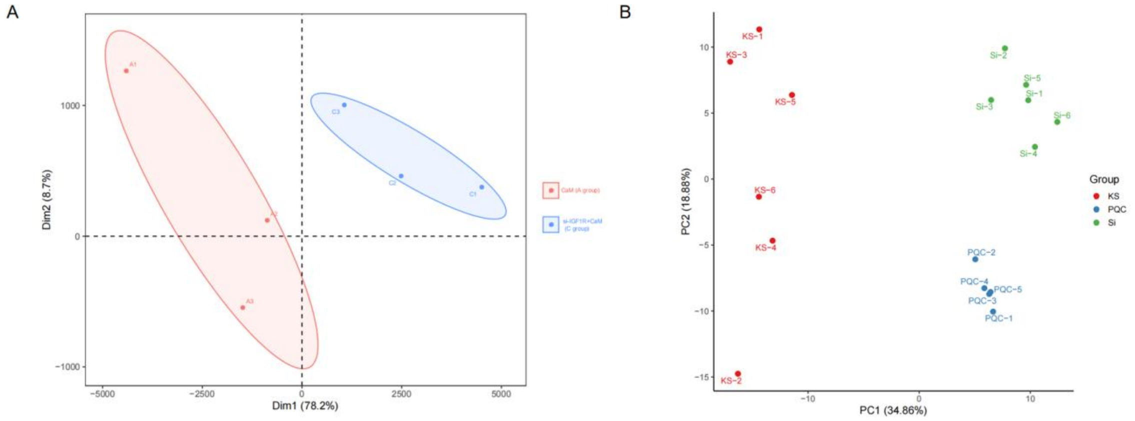

Figure S3 The mRNA trends of glycolysis pathway-related proteases in calcium oxalate crystal-induced cell injury. HK2: Hexokinase, ENO1: Enolase 1, non-neuron, GPI: Phosphoglucose Isomerase, ALDOA: Aldolase, fructose-bisphosphate A, and PGK1:Phosphoglycerate Kinase-1

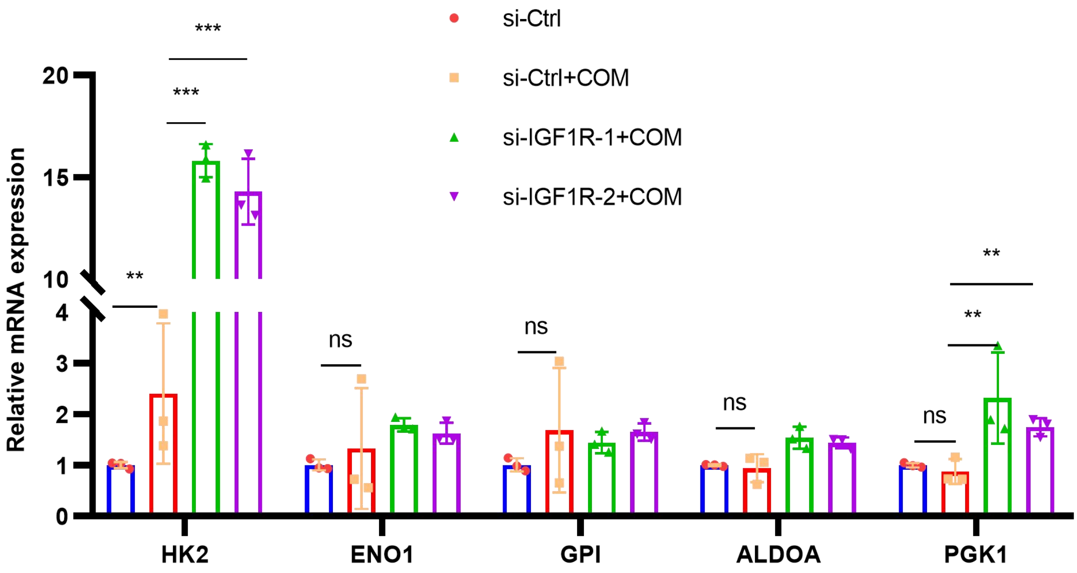

Supplementary Table S1 Primers of qRT-PCR used in this study

	Sequence of oligos used for quantitative real-time PCR of human genes	
	**Forward primer**	**Reverse primer**
β-ACTIN	CCT GGC ACC CAG CAC AAT	GGG CCG GAC TCG TCA TAC
IGF1R	CAG AGG AGC TGG AGA TGG AG	TCT CAG CCT TGT GTC CTG AG
CDH1	CGA GAG CTA CAC GTT CAC GG	GGG TGT CGA GGG AAA AAT AGG
CDH2	AGC CAA CCT TAA CTG AGG AGT	GGC AAG TTG ATT GGA GGG ATG
VIMENTIN	AGT CCA CTG AGT ACC GGA GAC	CAT TTC ACG CAT CTG GCG TTC
SNAIL	TCG GAA GCC TAA CTA CAG CGA	AGA TGA GCA TTG GCA GCG AG
IL-1β	ATG ATG GCT TAT TAC AGT GGC AA	GTC GGA GAT TCG TAG CTG GA
IL-6	ACT CAC CTC TTC AGA ACG AAT TG	CCA TCT TTG GAA GGT TCA GGT TG
IL-10	GAC TTT AAG GGT TAC CTG GGT TG	TCA CAT GCG CCT TGA TGT CTG
TNF-α	GAG GCC AAG CCC TGG TAT G	CGG GCC GAT TGA TCT CAG C
IGF2R	CAC CAA GTA GGC ACC ACT AAG	CAC CAA GTA GGC ACC ACT AAG
MCP-1	CAG CCA GAT GCA ATC AAT GCC	TGG AAT CCT GAA CCC ACT TCT
CLCX1	CTG CTC CTG CTC CTG GTA G	AGT GTG GCT ATG ACT TCG GT
CL3CX1	GCC ACA GGC GAA AGC AGT A	GGA GGC ACT CGG AAA AGC TC
HK2	GAG CCA CCA CTC ACC CTA CT	CCA GGC ATT CGG CAA TGT G
GPI	CAA GGA CCG CTT CAA CCA CTT	CCA GGA TGG GTG TGT TTG ACC
ALDOA	ATG CCC TAC CAA TAT CCA GCA	GCT CCC AGT GGA CTC ATC TG
ENO1	AAA GCT GGT GCC GTT GAG AA	GGT TGT GGT AAA CCT CTG CTC
PGK1	TGG ACG TTA AAG GGA AGC GG	GCT CAT AAG GAC TAC CGA CTT GG
STAT3	CAG CAG CTT GAC ACA CGG TA	AAA CAC CAA AGT GGC ATG TGA
JAK2	CTT TGC CCT GTA TGA CGA GAA C	ACC TCA TCC GGT AGT GGA GC
SLC2A1	ACA GCG TTG ATG CCA GAC AG	GGC CAA GAG TGT GCT AAA GAA
PFKP	ACC TCC AGA ACG AAG GTC CTC	CGC CTA CCT CAA CGT GGT G
PFKM	AAG CAT CAT CGA AAC GCT CTC	GGT GCC CGT GTC TTC TTT GT
PFKL	CCT CTC ACA CAT GAA GTT CTC C	GTA CCT GGC GCT GGT ATC TG
PFKFB3	TTG GCG TCC CCA CAA AAG T	AGT TGT AGG AGC TGT ACT GCT T
PKM	TGG GTG GTG AAT CAA TGT CCA	ATG TCG AAG CCC CAT AGT GAA
LDHB	TGG TAT GGC GTG TGC TAT CAG	TTG GCG GTC ACA GAA TAA TCT TT
GAPDH	AAA AGC GGG GAG AAA GTA GG	AAG AAG ATG CGG CTG ACT GT
LDHA	ATG GCA ACT CTA AAG GAT CAG C	CCA ACC CCA ACA ACT GTA ATC T
LDHA site 1	AGC CAA CTT CAG CTC TCT G	AGG TTC TGA AAT GGG GCT C
LDHA site 2	AGA GCC CCA TTT CAG AAC CT	GTG CAG TGA CTC ATG CCT GT
LDHA site 3	GGC TCC TTC CTG AGG CTA TC	TCT GGG CCT GTA TTC TTG CT

Supplementary Table S2 Primers of qRT-PCR used in this study

	Sequence of oligos used for quantitative real-time RT-PCR of mouse genes	
	**Forward primer**	**Reverse primer**
β-Actin	GGC TGT ATT CCC CTC CAT CG	CCA GTT GGT AAC AAT GCC ATG T
IGF1R	GTG GGG GCT CGT GTT TCT C	GAT CAC CGT GCA GTT TTC CA
CDH1	CAG TTC CGA GGT CTA CAC CTT	TGA ATC GGG AGT CTT CCG AAA A
CDH2	AGG CTT CTG GTG AAA TTG CAT	GTC CAC CTT GAA ATC TGC TGG
Vimentin	CGT CCA CAC GCA CCT ACA G	GGG GGA TGA GGA ATA GAG GCT
Snail	CAC ACG CTG CCT TGT GTC T	GGT CAG CAA AAG CAC GGT T
IL-1β	GCA ACT GTT CCT GAA CTC AAC T	ATC TTT TGG GGT CCG TCA ACT
IL-6	TAG TCC TTC CTA CCC CAA TTT CC	TTG GTC CTT AGC CAC TCC TTC
MCP-1	AAG CTT CGC CCC ACA TGC CTC	TCT CGA GCT CAC GCG CAG G
IL-10	GCT CTT ACT GAC TGG CAT GAG	CGC AGC TCT AGG AGC ATG TG
TNF-α	CCCTCACACTCAGATCATCTTCT	GCT ACG ACG TGG GCT ACA G

Supplementary Table S3 Details of antibodies

**Antibodies**	**Company**	**Cat #**	**Dilution**
IGF1R	GenTex	GTX637795	1:2000
IGF2R	Origene	TA351279	1:1000
E-cadherin	Affinity	AF0131	1:1000
N-cadherin	Origene	TA503933	1:1000
Vimentin	Origene	TA801297S	1:1000
Snail	Origene	TA500366	1:1000
STAT3	Cell Signaling Technology	9139	1:2000
Phospho-Stat3	Cell Signaling Technology	9145	1:2000
JAK2	ABclonal	A7694	1:1000
Phospho-JAK2	ABclonal	AP0531	1:1000
LDHA	Origene	TA500531	1:800
KIM-1	Abmart	MA8164S	1:1000
beta Actin	Affinity	AF7018	1:2000
Goat Anti-Rabbit IgG H&L（HRP）	Zenbio	511203	1:10000
Goat Anti-mouse IgG H&L（HRP）	Zenbio	511103	1:10000
Goat Anti-Rabbit IgG H&L（HRP）	Proteintech	30000-0-AP	1:5000

Supplementary Table S4 siRAN and shRNA used in this study

	Sense（5’-3’）
si-NC	UUCUCCGAACGUGUCACGUTT
si-IGFR-1	GCGGUGUCCAAUAACUACAUU
si-IGFR-2	TCATCAGCTTCACCGTTTA
si-STAT3	GCTGACCAACAATCCCAAGAA
si-LDHA	GCGTAACGTGAACATATTTAA
sh-IGF1R	CCAACGAGCAAGTTCTTCGTTtagtgaagccacagatgtaAACGAA GAACTTGCTCGT TGG

Supplementary Table S5 Other reagents

Product Name	Company	Cat #	Usage
Calcium Oxalate Monohydrate (COM)	Sigma	5794-28-5	Mix with complete culture medium, and add 200 ng of the reagent to each well of a 6-well plate
Glyoxylic acid(Gly)	Sigma	G10601	Dilute with physiological saline and administer intraperitoneally to mice at a concentration of 100 mg/kg
Stattic	TargetMol	T6308	Stattic was used in 5 μM to inhibit the activation of STAT3 after we confirmed it had no effect on cell viability of HK-2 cell
Colivelin	TargetMol	TP1856	For *in vivo* experiments, use a concentration of 1 mg/kg with a dissolution solution of 5% DMSO, 40% PEG300, 5% Tween 80, and 50% physiological saline, and perform continuous injections for 6 days. In *in vivo* experiments, use a concentration of 10 μM with a duration of 12 hours
Picropodophyllotox in (PPP）	MedChem Express	HY-15494	For *in vivo* experiments, use a concentration of 20 mg/kg, administer intraperitoneally twice daily, and continue for 7 days
2-deoxyglucose (2-DG）	MedChem Express	HY-13966	Pre-treat by adding the reagent 3 hours before adding calcium oxalate crystals, then add it together with calcium oxalate crystals to HK2 cells and replace the medium after 24 hours
2-NBDG	MedChem Express	HY-11621 5	Remove the cell culture medium and add fresh medium containing 2-NBDG (10 μM). Incubate at 37°C for 60 minutes.
